# Power-oriented resistance training combined with high-intensity interval training in pre-frail and frail older people: comparison between traditional and cluster training set configurations on the force–velocity relationship, physical function and frailty

**DOI:** 10.1007/s00421-023-05298-x

**Published:** 2023-09-09

**Authors:** Ivan Baltasar-Fernandez, Julian Alcazar, Susana Martín-Braojos, Ignacio Ara, Luis M. Alegre, Francisco José García-García, Ana Alfaro-Acha, José Losa-Reyna

**Affiliations:** 1 GENUD Toledo Research Group, Universidad de Castilla-La Mancha, Toledo, Spain; 2 CIBER of Frailty and Healthy Aging (CIBERFES), Madrid, Spain; 3 Faculty of Health Sciences, Universidad de Castilla-La Mancha, Talavera de la Reina, Spain; 4 Department of Geriatrics, Complejo Hospitalario de Toledo, Hospital Virgen del Valle, Toledo, Spain; 5 Grupo de Investigación Valoración del Rendimiento Deportivo, Actividad Física y Salud y Lesiones Deportivas (REDAFLED), Universidad de Valladolid, Calle Universidad S/N, 42004 Soria, Spain

**Keywords:** Muscle power, Cluster training, Resistance training, High-intensity interval training, Intra-set rest

## Abstract

**Objectives:**

To analyse the force–velocity relationship changes in response to two different training programmes differing in the set configuration (cluster *vs.* traditional), and their impact on physical function and frailty in pre-frail and frail older adults.

**Methods:**

43 pre-frail and frail (Frailty Phenotype ≥ 1 criteria) older adults (81.4 ± 5.1 years) participated in this study. Participants were assigned to cluster (CT; *n* = 10; 10-s intra-set rest), traditional (TT; *n* = 13; no intra-set rest) or control (CON; *n* = 20) groups. Force–velocity relationship (F_0_, V_0_ and P_max_), physical function (Short Physical Performance Battery, SPPB) and frailty (Frailty Phenotype, FP) were assessed at baseline and after the training programme.

**Results:**

Both CT and TT groups showed similar improvements in P_max_ after training (CT =  + 36.7 ± 34.2 W; TT =  + 33.8 ± 44.6 W; both *p* < 0.01). V_0_ was improved by both CT (+ 0.08 ± 0.06 m s^−1^; *p* < 0.01), and TT (+ 0.07 ± 0.15 m s^−1^, *p* > 0.05). F_0_ remained unchanged in CT (+ 68.6 ± 224.2 N,* p* > 0.05) but increased in TT (+ 125.4 ± 226.8 N,* p* < 0.05). Finally, SPPB improved in both training conditions (CT =  + 2.3 ± 1.3 points; TT =  + 3.0 ± 1.2 points; both *p* < 0.05) and in the CON group (+ 0.9 ± 1.4 points, *p* < 0.05). CT and TT reduced their FP (CT = − 1.1 criteria; TT = − 1.6 criteria; both *p* < 0.01), while no changes were observed in the CON group (− 0.2 criteria, *p* = 0.38).

**Conclusions:**

Both training methods were equally effective for improving P_max_, physical function and reducing frailty in pre-frail and frail older people. TT may be effective for improving both force and velocity parameters, while CT may be effective for improving velocity parameters alone, although further research is required to confirm these findings.

## Introduction

Health-related changes associated with ageing include an increase in morbidity and a decline in functional performance, which are two separate conditions despite being connected. Thus, as people age, preserving physical function is a key factor in preserving quality of life and reducing the risk of numerous adverse events, which translates into a greater number of years lived free of diseases and/or syndromes (i.e. frailty) (WHO 2016). Pre-frailty or frailty affects 50% of community-dwelling older people over 65 years (Garcia-Garcia, et al. 2011) and is clinically relevant because it is associated with disability as a result of loss of functional status and physiological reserve (Fried et al. 2001). As a matter of fact, frailty negatively affects multiple physical tasks like gait and mobility, balance, aerobic endurance and muscle strength. It is crucial to remember, though, that frailty can be reversed with certain therapies, particularly in the early phases (Morley et al. 2013). Therefore, preventing, detecting and treating frailty is an important public health challenge not only to enhance the quality of life for elderly, but also to minimise healthcare costs (Lutz et al. 1997).

Older adults’ functional capacity is significantly influenced by the neuromuscular system. The most significant neuromuscular result related with functional limitations and disability as people age is thought to be decreasing muscle power (Byrne et al. 2016). Since muscle power is defined as the product of force and velocity, an impaired muscle power can be caused by either force or velocity deficits in older adults (Alcazar et al. 2018a). In other words, two persons may have the same power output while different force and velocity capacities. The force component of muscle power is strongly associated with skeletal muscle size and single fibre specific force (or force per unit cross-sectional area), which is also influenced by other factors such as muscle architecture and the infiltration of fat and connective tissue into the skeletal muscle mass (Raj et al. 2010). On the other hand, tendon characteristics, fibre type and length seem to have a greater impact on the velocity component of muscle power (Roie et al. 2020). Additionally, related to physical function, older individuals may have neuromuscular characteristics underpinning movement velocity that are more degraded than those underlying force production (after taking into account muscle size) (Pojednic et al. 2012). Being a critical determinant of power output and functional ability in older adults, particularly in those with mobility limitations (Pojednic et al. 2012), movement velocity is a greater predictor of performance in lower-intensity functional tasks than muscle strength (Sayers et al. 2005).

Concurrent training (a combination of strength and aerobic training) is considered a cornerstone to improve functionality, cardiorespiratory and neuromuscular parameters in (pre)frail older adults (Cadore et al. 2013). High-intensity interval training (HIIT) appears to be an alternative and time-efficient solution to traditional aerobic training. It produces physiological advantages comparable to those of traditional aerobic exercise in less time by alternating short bursts of high intensity with prolonged intervals of reduced intensity for recovery (Gillen and Gibala 2014).

Interestingly, skeletal muscle power rather than strength is more significantly related with functional performance in older adults (Byrne et al. 2016). Consequently, resistance training (RT), with a particular emphasis on high-speed movement, appears to be a crucial component of a concurrent exercise programme (Pojednic et al. 2012). Keeping this in mind, it is important to note that the adaptations produced by RT may vary depending on training volume, load, velocity, training frequency, work/rest ratio or set configuration, among others. The set configuration, which is determined by how many repeats are executed in a set in relation to the maximum number of repetitions that can be performed, was the focus of this study (Iglesias-Soler et al. 2016). Thus, control over the number of repetitions in a set as well as the rest period of time between repetitions has proved to be an effective way to induce different types of neuromuscular adaptations (Iglesias-Soler et al. 2017; Rial-Vázquez, et al. 2020). A recent study conducted in young people suggests that the introduction of short inter-repetition rest periods (i.e. cluster set) may result in a greater increase in velocity-related parameters compared to the traditional configuration (Rial-Vázquez, et al. 2020). As mentioned above, when performing various functional tasks, the velocity component of muscle power is thought to be a crucial factor in older people (Pojednic et al. 2012). In this sense, improvements on velocity are likely to lead to improvements in physical function and reduce frailty status.

However, to the authors’ knowledge, no research have analysed the effect of cluster set configuration on the force–velocity (F–V) relationship and functional capacity in older people with (pre)frailty. Therefore, the main goal of this study was to analyse the changes in lower-limb F–V relationship after a cluster or traditional set configuration RT programme in older adults. Second, this study aimed to examine the impact of both cluster and traditional set configurations on physical function, frailty, and disability in activities of daily living.

## Methods

### Study design

To address the purposes of this study, a quasi-experimental non-randomised single-blinded controlled study was conducted. To compare the effects of concurrent training using different RT set configurations (traditional *vs.* cluster) on the F–V relationship and functional performance, three groups were created [cluster training (CT), traditional training (TT) and control group (CON)]. Randomisation was not possible due to ethical issues (Izquierdo et al. 2016) and the exercise intervention was proposed to all participants. Baseline evaluation was carried out before group allocation and those older adults who declined to take part in the intervention or could not come due to transport issues, were allocated to CON. Older adults who agreed to participate were allocated to either training group (TT or CT). Two training blocks were carried out, first the TT for which 20 participants were recruited. Subsequently, 11 individuals were recruited for CT, including cardiac patients. Prior to the start of the training programme, both training groups performed two familiarisation sessions with the training equipment. The F–V relationship was assessed on a different day. The participants of the CON group were advised not to change their eating or physical activity habits during the course of the study. All participants underwent a second evaluation at the conclusion of the training period. The F–V relationship test was carried out by CT and TT subjects 48 h following the final evaluation.

### Participants

Eighty-three older adults (45 women and 38 men; mean age: 81.4 ± 5.1 years old) were recruited at the Frailty Unit of the *Hospital Virgen del Valle* that belongs to the *Complejo Hospitalario* of Toledo, Spain. In the first concerted visit, a geriatrician rated the patient’s eligibility, who was invited to participate in the study if the patient met criteria. Inclusion criteria were: (a) being 75 years of age or older; (b) diagnosed as pre-frail or frail according to the Frailty Phenotype (Fried et al. 2001); (c) having a score between 2 and 9 points (both included) on the Short Physical Performance Battery (SPPB) (Guralnik et al. 1994); and (d) being able to walk independently or assisted. Additionally, exclusion criteria included severe cognitive impairment [Mini-mental state examination (MMSE) < 18] (Tombaugh and McIntyre 1992), severe disability [score < 15 points on Barthel index (Mahoney and Barthel 1965)]. All participants gave informed consent, and the study was conducted in accordance with the Helsinki Declaration of 1975, as last modified in 2000, on the conduct of clinical research, and was approved by the Ethical Committee of the Toledo Hospital.

### Sociodemographic, vital signs and anthropometric data

On the first day of assessment, sociodemographic data such as age and sex were collected. In addition, heart rate, blood pressure (M2, OMROM, Japan) and percentage oxygen saturation (CMS50F, Contect Medical Systems, China) at rest were assessed.

Height and body mass were collected using a stadiometer with a precision of 1 mm (Seca 711, Hamburg, Germany) and a scale device with a precision of 100 g (Seca 711, Hamburg, Germany), respectively. Then, body mass index (BMI) was calculated as body mass divided by height^2^ (kg m^−2^).

### Frailty status

Frailty Phenotype (Fried et al. 2001) and Frailty Trait Scale Short Form (FTS-5) (García-García et al. 2020) were used to assess frailty. Briefly, Frailty Phenotype evaluates 5 criteria, including habitual gait speed, handgrip strength (HS) (Jamar Preston, Jackson, MI, USA), involuntary weight loss, exhaustion and low physical activity measured through the “Physical Activity Scale for the Elderly” (PASE) (Washburn et al. 1993). Participants were classified as frail, pre-frail or robust depending on the number of criteria they presented (3–5, 1–2 or 0 criteria, respectively) (Fried et al. 2001). On the other hand, FTS-5 evaluates 5 domains, including BMI, physical activity, static balance, habitual gait speed and HS. Each of the FTS-5 components provides a score ranging from 0 to 10 points (i.e. final score between 0 and 50 points). Participants who scored 25 points or less were classified as robust, while patients who scored over 25 points were classified as frail (García-García et al. 2020).

### Physical function

Physical function was evaluated using the SPPB, which includes static balance in three different positions (feet together, semi-tandem and full tandem), habitual gait speed in 4 m and the 5 repetitions sit-to-stand test (Guralnik et al. 1994). Additionally, maximal gait speed (MGS) was measured using the 10-m walking test (Rossier and Wade 2001). Participants were instructed to walk the 10-m distance at their maximum possible speed without running. Two tries were performed, and the best time was recorded and used to determine MGS by dividing the distance travelled by the time needed to complete the test.

### F–V relationship and STS power assessment

F–V relationship was evaluated in TT and CT participants after performing two separate familiarisation sessions with the leg press equipment (Element + Inclusive, Technogym, Barcelona, Spain). Procedures detailed elsewhere were followed (Alcazar et al. 2017). Briefly, after a warm-up on a treadmill (Lode BV, Valiant 2 Rehab, Groningen, Netherlands) and on the leg press, participants performed two sets of one repetition with gradually increasing loads (10-kg increments) from 40% of their body mass. Joint angles of knee and hip were standardised to ensure the same starting position of all the participants (90º and 70º, respectively; full extension = 180º). Mean force and velocity data during the concentric phase of each repetition were recorded against at least 3 loads with a linear position transducer with a 1000-Hz sample rate (T-Force System, Ergotech, Murcia, Spain). All participants were verbally encouraged to perform the concentric phase as fast and strong as possible, whereas self-elected eccentric velocity was demanded to achieve the maximal performance in the following repetition. Moreover, sufficient rest was allowed between each repetition (based on the velocity of the preceding repetition). A linear model was fitted to the F–V data collected and the theoretical maximal isometric force (F_0_) and maximal unloaded velocity (V_0_) were obtained from the force and velocity intercepts, respectively. Then, maximal muscle power [P_max_ = (F_0_ · V_0_)/4] and the optimal load at which the subjects exerted their maximum power were calculated.

As a matter of fact, in order to compare the changes in muscle power of CT and TT with respect to CON, mean mechanical power was estimated from the 5 repetition sit-to-stand test using a previously validated equation (Alcazar et al. 2018b):

$$\mathrm{STS\, power} \left(W\right)=\frac{\mathrm{Body \,mass}\, \left(kg\right)\times 0.9\times g\times \left[\mathrm{Height} \left(m\right)\times 0.5-\mathrm{Chair \,height} \left(m\right)\right]}{\left[\frac{\mathrm{STS\, time} \left(s\right)}{\mathrm{n \,of \,reps}}\right]\times 0.5}.$$

Finally, STS power normalised by body mass (i.e. relative STS power) was calculated, as it has been shown to be a stronger predictor of functional impairment than absolute STS power (Alcazar et al. 2018b).

### Concurrent training protocol

Both CT and TT performed the same concurrent training programme described elsewhere (Losa-Reyna et al. 2019). Briefly, they performed 2 training sessions per week (≈ 45 min/session) for 6–8 weeks that consisted of RT (leg press and plantar flexion exercises) and cardiovascular exercise on a treadmill. Regarding to RT, participants performed 3–4 sets of 8–14 repetitions at 30–60% of the estimated maximal isometric force (F_0_). The concentric phase of each repetition was performed as fast as possible to favour power production in both groups. The only difference between training groups was the set configuration. Specifically, CT had a 10-s rest every 2 repetitions, while TT had no intra-set rest. Both groups rested 1 min between sets. Cardiovascular training was based on high-intensity interval training (combining habitual and maximal gait speed). The first two weeks consisted of 8–10 min of continuous exercise at 50% of MGS and also served as conditioning. The following weeks, the HIIT protocol consisted in 6–10 intervals with a work:rest ratio of 1:5 (10–20 s at 90% of MGS and 50–100 s at 50% UGS) increasing to a 1:3 ratio (30 s at 90% MGS and 90 s at 50% UGS) at the end of the programme. Percentage of adherence was calculated by dividing the number of sessions prescribed by the number of sessions completed and multiplying by 100.

### Statistical analysis

Data have been presented as mean and standard deviation. Proportions were used to summarise discrete variables. One way-ANOVA was used to assess statistical differences between TT, CT and CON at baseline. Mixed model repeated measures ANOVA adjusted for baseline values and weeks of training completed was conducted to compare the effects of each set configuration. A Bonferroni’s post hoc test was conducted when between-group differences were observed. To determine the magnitude and the meaningfulness of the findings, Cohen’s *d* was calculated, and the following cut-offs were applied to determine the magnitude and meaningfulness of changes: small (from 0.2 to 0.5), medium (from 0.5 to 0.8), or large (over 0.8) (Cohen 2013). All data were analysed with SPSS Statistics 22.0 (SPSS Inc., Chicago, IL, USA) for Windows and the level of significance was established at the level of *p* < 0.05.

## Results

**Fig. 1:**
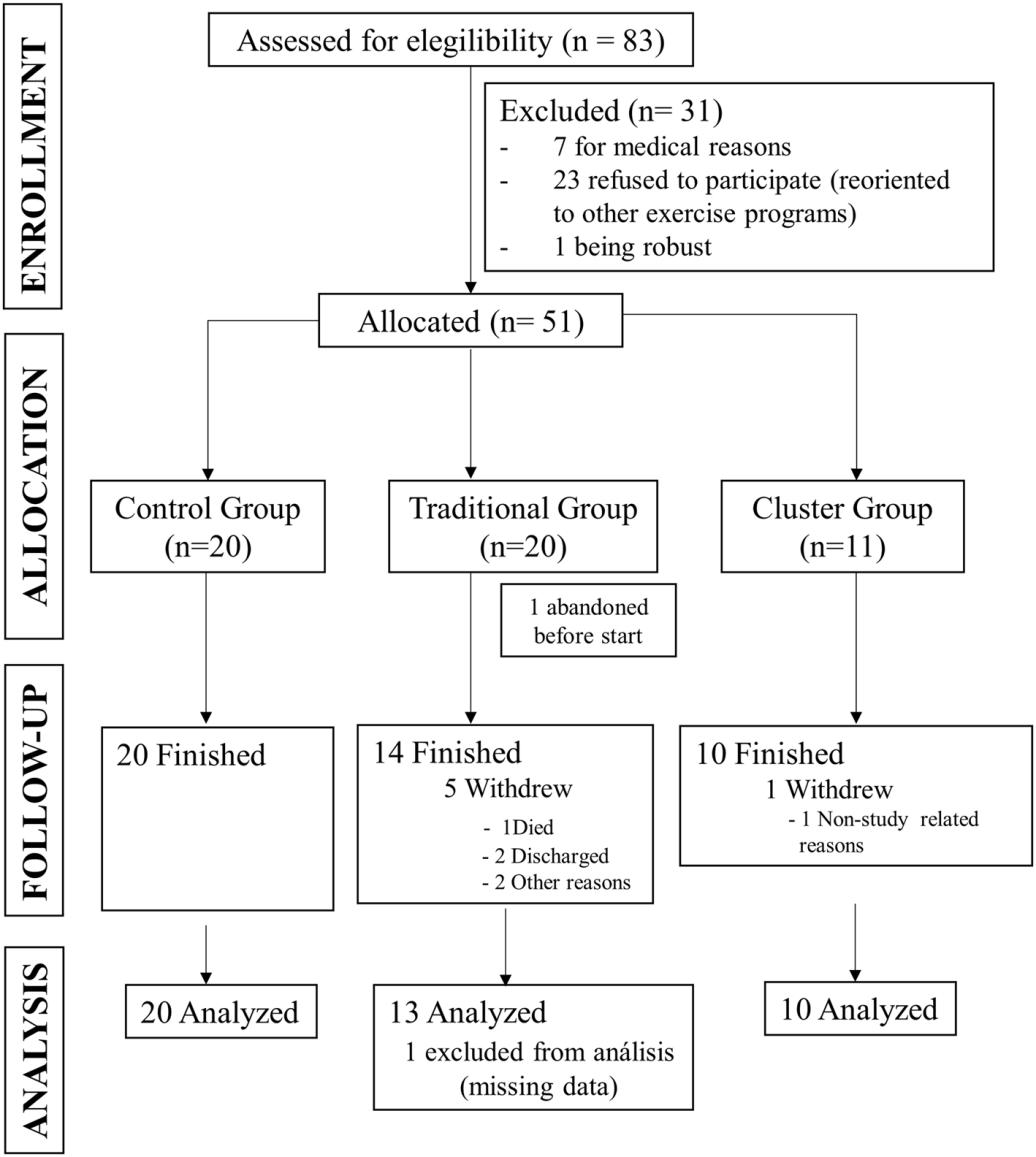
CONSORT diagram with participant flow

We screened a total of 83 participants of whom 51 were allocated (Fig. 1). Twenty were allocated to CON, twenty to TT and eleven to CT. One participant in TT abandoned before the beginning of the intervention and 5 participants withdrew from the study while only 1 participant had to withdraw in the CT group. Finally, 43 participants took part in the study, 13 in TT, 10 in CT and 20 in CON (Fig. 1). Adherence was similar in TT and CT groups (90.% and 90.%, respectively; *p* = 0.998).

### Baseline characteristics

Baseline characteristics of the participants are shown in Table 1. A total of 26 (60%) participants were frail, and 17 (40%) pre-frail according to the Frailty Phenotype. Even though all participants were frail or pre-frail, they preserved a high level of independence in basic activities of the daily living (Barthel index = 87.33 ± 12.69 points). There were no significant differences between groups at baseline (Table 1).

**Table 1:** Baseline characteristics of the study participants

	Control	Traditional training	Cluster training
	(*N* = 20)	(*N* = 13)	(*N* = 10)
Age (years)	82.6 ± 5.4	82.6 ± 6.2	79.2 ± 3.9
Women, *N* (%)	16 (80)	9 (69.2)	6 (50.0)
Height (cm)	154.2 ± 7.3	155.8 ± 9.9	162.4 ± 10.8
Weight (kg)	70.8 ± 17.0	66.3 ± 12.1	73.8 ± 15.8
BMI (kg m^−2^)	29.8 ± 6.8	27.2 ± 3.9	27.9 ± 5.0
Systolic pressure (mmHg)	142.5 ± 18.5	137.1 ± 21.6	141.4 ± 19.7
Diastolic pressure (mmHg)	73.7 ± 15.5	75.4 ± 8.9	72.5 ± 14.1
Heart rate (bpm)	75.6 ± 12.1	74.9 ± 14.2	69.3 ± 7.0
SpO2 (%)	96.0 ± 2.4	95.5 ± 2.3	96.0 ± 1.7
Barthel index (points)	82.8 ± 15.6	90.4 ± 8.4	92.5 ± 7.9
Frailty phenotype	3.1 ± 1.4	3.2 ± 1.1	2.2 ± 0.9
FTS-5	25.7 ± 6.1	24.1 ± 6.1	21.9 ± 4.4

*BMI* body mass index, *SpO2* percentage oxygen saturation, *FTS-5* frailty trait scale short form

^*^Significant differences compared to the CON group (*p* < 0.05)

^#^Significant differences compared to traditional training group (*p* < 0.05)

### Effects of the intervention on physical function

The effects of the intervention on physical function parameters are shown in Table 2. TT, CT, and CON improved their SPPB score (TT: 43.5%; CT: 31.1%; CON: 13.4%) and UGS (TT: 26.8%; CT: 31.7%; CON: 9.1%) after the intervention. There were no significant differences between TT and CT groups in terms of SPPB (*p* = 0.558) or UGS changes (*p* = 0.999); however, there were differences between TT and CT groups compared to the CON group in terms of SPPB (*p* < 0.001 and *p* = 0.017, respectively) and UGS changes (*p* = 0.004 and *p* < 0.001, respectively). Balance time was improved in the TT group (3.3%; *p* < 0.05) but not in the CT or CON groups (CT: 1.2%; CON: − 0.4%; both *p* > 0.05). No differences between TT, CT and CON groups were found.

**Table 2:** Effects of the set configuration on physical function and STS power adjusted by baseline values and weeks of training

	Control (*N* = 20)			Traditional (*N* = 13)			Cluster (*N* = 10)		
	Baseline	Change		Baseline	Change		Baseline	Change	
	Mean ± SD	Δ ± SD	Cohen’s *d*	Mean ± SD	Δ ± SD	Cohen's *d*	Mean ± SD	Δ ± SD	Cohen’s *d*
SPPB score (points)	6.7 ± 2.2	0.9 ± 1.4*	0.43	6.9 ± 1.8	3.0 ± 1.2*^,#^	1.44	7.4 ± 2.4	2.3 ± 1.3*^,#^	1.13
Balance (s)	24.4 ± 5.3	− 0.1 ± 0.8	0.02	24.4 ± 5.5	0.8 ± 0.9*	0.16	25.8 ± 4.0	0.3 ± 1.3	0.06
UGS (m s^−1^)	0.55 ± 0.20	0.05 ± 0.09*	0.26	0.56 ± 0.18	0.15 ± 0.09*^,#^	0.78	0.60 ± 0.18	0.19 ± 0.08*^,#^	0.98
Absolute STS Power (W)	144.5 ± 51.8	25.6 ± 25.0*	0.51	131.9 ± 38.4	59.2 ± 30.4^*,#^	1.18	188.9 ± 45.4	57.7 ± 25.4*^,#^	1.15
Relative STS Power (W kg^−1^)	2.0 ± 0.5	0.4 ± 0.3*	0.85	2.0 ± 0.3	0.8 ± 0.3*^,#^	1.70	2.6 ± 0.3	0.8 ± 0.4*^,#^	1.70

*SPPB* short physical performance battery, *UGS* usual gait speed (4 m), *STS* sit-to-stand

*Significant change (*p* < 0.05)

^#^Significantly different compared to control group (*p* < 0.05)

### Effects of the intervention on frailty status

**Fig. 2:**
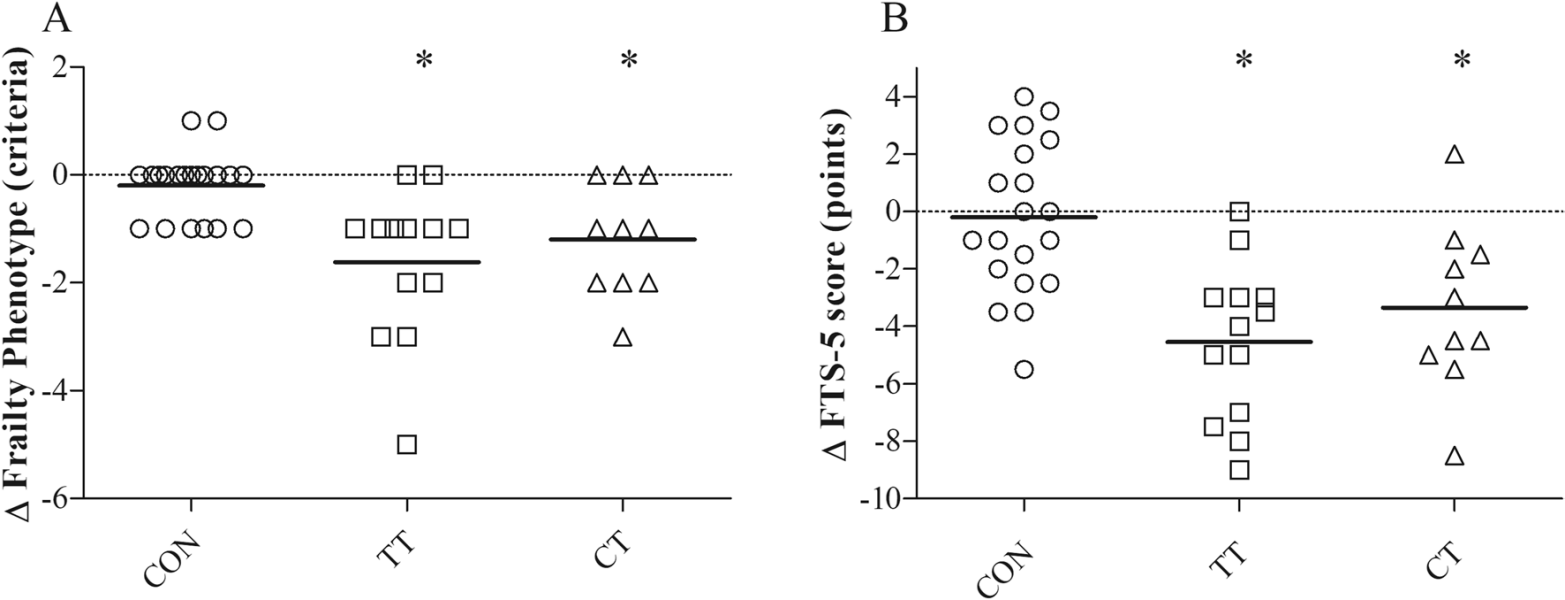
Individual changes in Frailty Phenotype (**A**) and FTS-5 (**B**) after the intervention. *Significantly different compared to CON (*p* < 0.05). Horizontal solid lines indicate the average change of each variable. *CON* control group, *TT* traditional training group, *CT* cluster training group

Both TT and CT reduced their Frailty Phenotype criteria after the intervention (TT: -1.6 ± 1.4 points, *d* = 1.29; CT: − 1.2 ± 1.0 points, *d* = 0.97;* p* < 0.05) while no changes were found in CON (− 0.2 ± 0.6,* d* = 0.16) (Fig. 2 A). Significant differences were found between CT and TT groups compared to the CON group in terms of Frailty Phenotype changes (*p* = 0.001 and *p* = 0.031, respectively). No differences were found between CT and TT groups (*p* = 0.933). Interestingly, a total of 8 (61.5%) and 5 (50%) participants improved their frailty status in TT and CT, respectively, whereas only 1 (4.7%) participant in CON improved his frailty status. According to FTS-5, both TT and CT reduced their frailty levels (TT: − 4.5 ± 2.7 points, *d* = 0.77; CT: − 4.5 ± 2.7 points, *d* = 0.77;* p* < 0.05), whereas no changes were found in CON (− 0.2 ± 2.7 points, *d* = 0.03,* p* > 0.05) (Fig. 2B). Significant differences were found between CT and TT groups compared to the CON group in terms of FTS-5 changes (both *p* < 0.001). No differences were found between CT and TT groups (*p* = 0.999). According to the FTS-5, out of 5 frail participants in the TT group and 3 in the CT group, 3 (60%) and 3 (100%) became robust after the intervention, respectively. This resulted in 85% of robust participants in the TT group and 100% in the CT group after the intervention. Regarding the CON group, out of 11 frail participants at baseline, 2 (18%) became robust after the intervention (55% of robust participants after the intervention).

### Effects of the intervention on STS power

TT, CT and CON improved both absolute STS power (TT: + 59.2 ± 30.4 W, *d* = 0.62; CT: + 57.7 ± 25.4 W, *d* = 0.69; CON: + 25.6 ± 25.0 W, *d* = 0.46; all *p* < 0.05) and relative STS power (TT: + 0.8 ± 0.3 W kg^−1^,* d* = 0.81; CT: + 0.8 ± 0.4 W kg^−1^, *d* = 0.89; CON: 0.4 ± 0.3 W kg^−1^, *d* = 0.62; all *p* < 0.05) after the intervention (Table 2). There were significant differences between TT and CT groups compared to the CON group in both absolute STS power (*p* = 0.005 and *p* = 0.024, respectively) and relative STS power changes (*p* = 0.001 and *p* = 0.015, respectively). There were no differences between the TT group and the CT group in absolute (*p* = 0.999) or relative STS power changes (*p* = 0.999).

### Effects of the intervention on the F–V relationship

The effects of the intervention on the F–V relationship are shown in Table 3. The intervention induced improvements in P_max_ in both TT and CT (TT: + 33.8 ± 44.6 W; *d* = 0.44; CT: + 36.7 ± 34.1 W, *d* = 0.49;* p* < 0.05). Regarding F_0_, a significant improvement was observed in TT (+ 125.4 ± 226.8 N, *d* = 0.23, *p* < 0.05), while no changes were observed in CT (+ 68.6 ± 224.2 N, *d* = 0.15). V_0_ was increased in both TT (+ 0.07 ± 0.15 m s^−1^, *d* = 0.55; *p* > 0.05) and CT (+ 0.08 ± 0.06 m s^−1^, *d* = 0.65;* p* < 0.05) groups. Moreover, TT increased optimal load (+ 6.4 ± 11.5 kg, *d* = 0.27, *p* < 0.05), while no changes were observed in CT (+ 1.3 ± 9.7 kg, *d* = 0.09; *p* > 0.05). No differences between configurations were found of any of these parameters (P_max_, V_0_, F_0_, optimal load and maximal load) (*p* > 0.05).

**Table 3:** Effects of the exercise intervention on the F–V relationship variables adjusted by baseline values and weeks of training

	Traditional training (*N* = 13)			Cluster training (*N* = 10)		
	Baseline	Change		Baseline	Change	
	Mean ± SD	Δ ± SD	Cohen’s *d*	Mean ± SD	Δ ± SD	Cohen’s *d*
F_0_ (N)	941.0 ± 497.5	125.4 ± 226.8*	*0.23*	1394.6 ± 540.4	68.6 ± 224.2	0.12
V_0_ (m s^−1^)	0.51 ± 0.14	0.07 ± 0.15*	*0.56*	0.48 ± 0.11	0.08 ± 0.06*	0.63
P_max_ (W)	118.9 ± 65.5	33.8 ± 44.6*	*0.43*	170.5 ± 87.8	36.7 ± 34.2*	0.47
Load P_max_ (kg)	36.4 ± 20.9	6.4 ± 11.5*	*0.27*	50.5 ± 27.6	1.3 ± 9.7	0.05

*F*_*0*_ force-intercept, *P*_*max*_ maximum power, *V*_*0*_ velocity intercept

*Significant change (*p* < 0.05)

## Discussion

The main conclusions of the current study were that the muscle power of pre-frail and frail older adults was increased in a similar manner by cluster and traditional set configurations. However, the F–V parameters responded differently based on the set configuration of the training programme. On the one hand, traditional set configuration improved both force- and velocity-related parameters. On the other hand, cluster set configuration elicited major improvements on the velocity-related parameters. Finally, both configurations have successfully improved physical function and reduced frailty similarly in pre-frail and frail older adults (Fig﻿. 3).

**Fig. 3:**
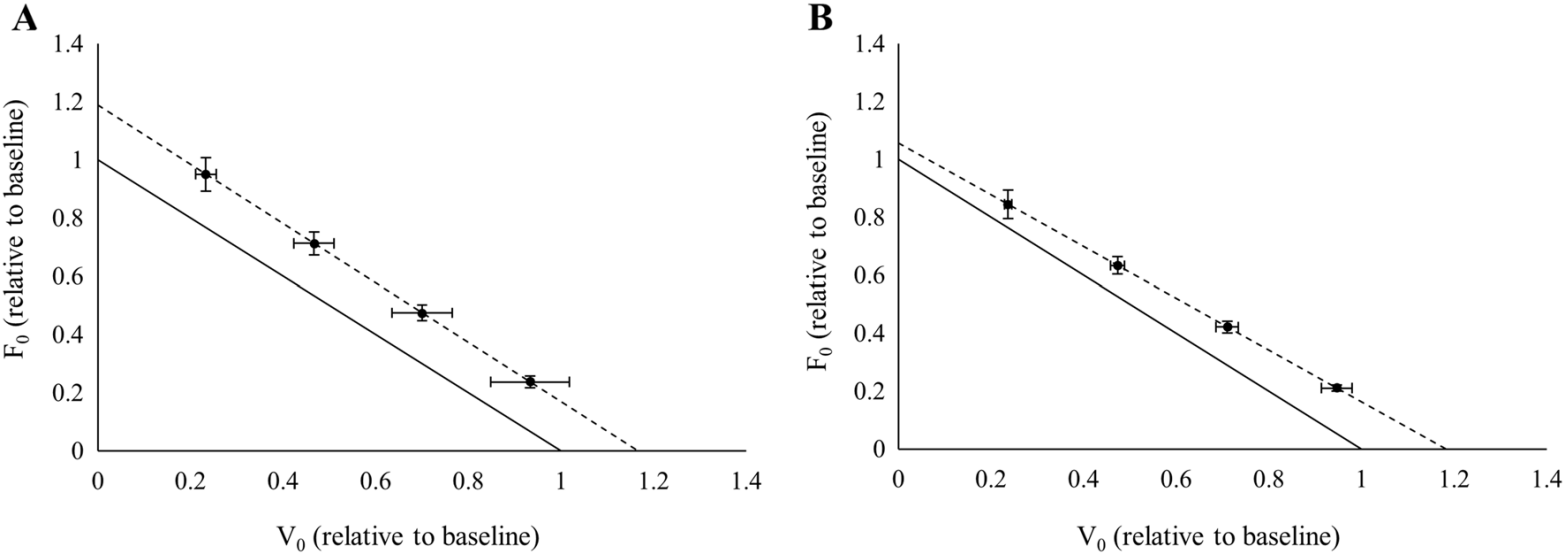
Changes in the force–velocity relationship yielded by TT (**A**) and CT (**B**). Solid lines show baseline values whilst dashed lines show post-training values. Data shown as relative to baseline levels (mean ± SD)

Rial-Vázquez et al. (2020) evaluated the effects of a RT programme with a traditional *versus* a cluster set configuration in active young people on the F–V relationship in two multi-joint exercises (bench press and parallel squat). They found that both CT and TT improved both F_0_ and V_0_ in the bench press while in the parallel squat, CT improved V_0_ and TT improved F_0_, with no differences between groups in either exercise. These results may have been possible due to a lower loss of velocity during training in the squat exercise, which would have allowed the subjects to train at higher relative velocities. However, we must be careful interpreting the results of Rial-Vázquez et al. since they were obtained from active young subjects. Overall, the results suggest that cluster training is an effective method for improving velocity parameters of muscle power (Iglesias-Soler et al. 2017; Rial-Vázquez et al. 2020). It is important to note that some studies in young people have not found differences in the F–V relationship after this type of training (Morales-Artacho et al. 2018). These discrepancies could be due to differences in training protocols (Morales-Artacho et al. 2018), the exercise performed (Iglesias-Soler et al. 2017; Rial-Vázquez, et al. 2020) or the characteristics of the study subjects. In our study, subjects trained on a leg press, a similar exercise compared to the squat (Sjöberg et al. 2020), and the loss of velocity could have been similar. Indeed, one of the factors that may have influenced the lack of improvement in F_0_ in the CT group, could have been the baseline values of F_0_, which, although not significantly different from the TT group, were 32.5% higher. Therefore, further studies are needed to determine whether cluster training indeed does not generate adaptations in F_0_. Nevertheless, our data suggest that traditional or cluster set configuration can similarly increase muscle power in older adults. This is of great relevance, since lower levels of muscle power are strongly associated with lower physical function (Byrne et al. 2016; Baltasar-Fernandez 2021; Losa-Reyna 2020), poorer quality of life (Baltasar-Fernandez 2021; Losa-Reyna 2020), frailty (Baltasar-Fernandez 2021; Losa-Reyna 2020), hospitalisation (Losa-Reyna et al. 2021) and even mortality (Losa-Reyna et al. 2021; Alcazar et al. 2021) in older adults.

In our study, physical function improved regardless of the set configuration used. A previous study showed that V_0_ was the only variable that was significantly associated with the ability to sit down and get up from a chair and the ability to climb stairs in older adults with mobility limitations, i.e. SPPB < 9 points (Pojednic et al. 2012). In our study, both TT and CT groups improved V_0_ similarly (+ 0.07 and + 0.08 ms^−1^, respectively), which translated to similar improvements in physical function as assessed by SPPB (+ 3.0 and + 2.3 points, respectively) and UGS (+ 0.15 and + 0.19 ms^−1^, respectively). Aside from velocity, muscle strength also contributes to physical function (Roie et al. 2011). However, a study comparing the effects of traditional and cluster set configuration concluded that, although both configurations were effective in improving physical function and quality of life, the cluster configuration led to significantly bigger improvements (Ramirez-Campillo et al. 2018). Differences could be explained by methodological disparities in training protocols. Unlike them, in our study the total rest between sets was not equalised. Since it is recognised that it may influence neuromuscular adaptations, the rest period between and within training sets is a crucial acute training variable that must be taken into account in RT prescription (Grgic et al. 2018). The type and characteristics of the training programme are key factors that could affect exercise-induced adaptations in frail older individuals (Cadore et al. 2013). Another factor that could explain these differences is the duration and frequency of the exercise programme.

In the study performed by Ramirez-Campillo et al. (2018), older women trained 3 times/week for 12 weeks; while in our study, participants performed twice a week for a maximum of 8 weeks. Compared to healthy older adults, frail older adults may require long-term exercise programmes with brief sessions (Cadore et al. 2013). Nevertheless, a 6-week exercise intervention has been demonstrated to be enough to improve physical function and frailty in frail older adults (Losa-Reyna et al. 2019).

In older people, biological age (i.e. frailty status) has a greater influence than chronological age on training-induced adaptations in older adults affected by frailty syndrome (Morley et al. 2013). Frailty has already been demonstrated to be reversible with proper exercise interventions (Cadore et al. 2013). To our knowledge, this is the first study that examines the effect of cluster set configuration in older adults with frailty or pre-frailty. Our results showed a reduction in frailty criteria regardless of the set configuration used. Although the 6–8-week training period produced significant improvements in frailty status, independently of the set configuration, prolonging the duration of the training programme could produce different results. To evaluate changes in frailty status, the use of a continuous scale like the FTS-5 may provide the ability to detect small changes due to is higher sensitivity. Moreover, the FTS-5 showed a stronger predictive ability for adverse events (i.e. hospitalisation, disability and mortality) than the Frailty Phenotype (García-García et al. 2020).

## Study limitations

The present study has a few limitations. First, F–V relationship, which was used for exercise prescription, was not evaluated in CON because these participants only assisted to the clinical evaluation visit, and it has been shown that a familiarisation period of at least two sessions is required before acquiring F–V data to minimise inaccuracies (Alcazar et al. 2018a, c, d). Due to the absence of a control group, these results should be interpreted with caution. Second, randomisation was not possible because participation in the exercise programme was offered to all participants and it would be unethical not to prescribe exercise to all participants, since exercise has already been shown to be effective and safe for frail older people (Izquierdo et al. 2016). Despite random allocation was not possible, baseline characteristics were similar between groups. Furthermore, baseline values were included as covariable in the statistical analysis performed. Several strengths should also be acknowledged (1) this is the first study to compare the effects of two different set configurations on the F–V relationship in frail older adults and (2) the use of cluster training has been demonstrated to be a safe training method in frail older adults when applied in the clinical setting, even with cardiac patients.

## Conclusion

Our findings suggest that different training configuration may lead to different adaptations on the F–V relationship in pre-frail and frail older adults. Specifically, traditional set configuration seems to be an effective option if the goal is to improve both force and velocity parameters, while cluster set configuration may be effective for improving velocity parameters alone. However, future studies are required to confirm the findings showed in our research. Furthermore, both methodologies improved maximum power in a similar way and were safe and similarly effective for improving physical function and reducing frailty.

## Data Availability

The findings of this study are supported by data accessible from the corresponding author upon a reasonable request.

## Abbreviations

BMI: Body mass index
CON: Control group
CT: Cluster training
F_0_: Theoretical maximal isometric force
FP: Frailty phenotype
FTS-5: Frailty trait scale short form 5 items
F–V: Force–velocity
HIIT: High-intensity interval training
MGS: Maximal gait speed
MMSE: Mini-mental state examination
PASE: Physical activity scale for the elderly
P_max_: Maximal power output
SPPB: Short physical performance battery
STS: Sit-to-stand test
TT: Traditional training
UGS: Usual gait speed
V_0_: Maximal unloaded velocity
